# Unveiling the Link Between Vitamin D, Hashimoto’s Thyroiditis, and Thyroid Functions: A Retrospective Study

**DOI:** 10.3390/nu17091474

**Published:** 2025-04-27

**Authors:** Rahime Evra Karakaya, Abbas Ali Tam, Pervin Demir, Gülsüm Karaahmetli, Sevgül Fakı, Oya Topaloğlu, Reyhan Ersoy

**Affiliations:** 1 Department of Nutrition and Dietetics, Faculty of Health Sciences, Ankara Yıldırım Beyazıt University, Ankara 06760, Türkiye; 2 Department of Endocrinology and Metabolism, Faculty of Medicine, Ankara Yıldırım Beyazıt University, Ankara 06800, Türkiye; aatam@aybu.edu.tr (A.A.T.); otopaloglu@ybu.edu.tr (O.T.); reyhanersoy@aybu.edu.tr (R.E.); 3 Department of Biostatistics and Medical Informatics, Faculty of Medicine, Ankara Yıldırım Beyazıt University, Ankara 06010, Türkiye; pervindemir@aybu.edu.tr; 4 Department of Endocrinology and Metabolism, Ankara Bilkent City Hospital, Ankara 06800, Türkiye; gulsumgedik85@gmail.com (G.K.); black_snowtr@yahoo.com (S.F.)

**Keywords:** vitamin D, thyroid autoimmunity, Hashimoto’s thyroiditis, free triiodothyronine (fT3), free thyroxine (fT4), thyroid-stimulating hormone (TSH)

## Abstract

Background/Objectives: Hashimoto’s thyroiditis (HT) is an autoimmune disease influenced by genetic factors and environmental triggers that affect immune system function. Data suggest that vitamin D may also play a role in the etiopathogenesis of HT. Methods: This retrospective study included patients admitted to the Endocrinology and Metabolic Diseases Outpatient Clinic. Data from individuals aged 18 years and older were analyzed, including serum levels of thyroid-stimulating hormone (TSH), free triiodothyronine (fT3), free thyroxine (fT4), anti-thyroid peroxidase (anti-TPO), anti-thyroglobulin (anti-TG), and vitamin D. HT was diagnosed based on the presence of anti-TPO and/or anti-TG antibodies, while individuals with negative results for both were classified as non-HT. Thyroid function was categorized as euthyroid if TSH levels were between 0.55 mU/L and 4.78 mU/L and fT4 levels were between 0.89 ng/dL and 1.76 ng/dL; hypothyroid status was defined as TSH > 4.78 mU/L. Vitamin D levels were classified as deficient (<50 nmol/L), insufficient (50–74.9 nmol/L), or sufficient (≥75 nmol/L). Results: Of the total participants, 25,018 did not have HT, while 27,800 were diagnosed with HT. Vitamin D level was significantly higher in the HT group than the non-HT group (41.43 nmol/L and 39.44 nmol/L, *p* < 0.001). Vitamin D deficiency was present in 65.5% of the non-HT group and 62.1% of the HT group (*p* < 0.001). Subgroup analyses based on thyroid function showed that vitamin D levels were highest in the euthyroid HT group and similar in the euthyroid non-HT, hypothyroid non-HT, and hypothyroid HT groups (*p* < 0.001). Conclusions: In conclusion, while vitamin D levels were higher in the HT group compared to the non-HT group, no clinically significant association between vitamin D levels and HT or autoantibody positivity was observed. Vitamin D deficiency was more prevalent in the hypothyroid group compared to the euthyroid group. This study suggests that although vitamin D deficiency may not be directly involved in the pathogenesis of HT, it may still play a role in modulating immune activity or influencing the disease phenotype..

## 1. Introduction

Vitamin D is essential not only for bone health but also for maintaining calcium and phosphorus balance in the body [1]. It has two main forms: vitamin D2 (ergocalciferol), derived from plant-based sources, and vitamin D3 (cholecalciferol), which is produced in the skin through exposure to sunlight [2]. Upon exposure to ultraviolet B radiation from sunlight, the skin synthesizes cholecalciferol from 7-dehydrocholesterol through the action of the enzyme 7-dehydrocholesterol reductase [3]. After synthesis, vitamin D undergoes 25-hydroxylation in the liver, resulting in the formation of 25-hydroxyvitamin D [25(OH)D], which is widely accepted as a marker of circulating vitamin D levels. Subsequently, 25(OH)D is delivered to the kidneys, where it is converted through an additional hydroxylation step, resulting in the formation of the biologically active compound 1,25-dihydroxyvitamin D (calcitriol) [1,25(OH)_2_D] [4].

Specifically, 1,25(OH)_2_D exerts its effects at the cellular level by binding to the vitamin D receptor (VDR), which belongs to the nuclear receptor superfamily and is expressed in nearly all tissues throughout the body. As a result, vitamin D has extensive effects on various organs and cells, such as bones, intestines, kidneys, pancreas, etc. [5]. Vitamin D plays a crucial role in maintaining calcium and phosphorus homeostasis in the intestines and kidneys. It also supports bone health by regulating mineralization and remodeling, ensuring proper growth and maintenance of the skeletal system [6].

In recent years, the health benefits of vitamin D have been the subject of much attention. In addition to its established role in bone and skeletal health, mounting evidence suggests that vitamin D may play a crucial role in the prevention of various conditions, including metabolic disorders [7], cardiovascular diseases [8], cancer [9], and immune-mediated diseases [10]. These effects extend to regulating various biological processes in the body through modulation at the genetic, immune, and cellular levels, primarily mediated by the VDR [11,12].

Vitamin D is essential for immune system function, contributing to both innate and adaptive immunity [13]. It suppresses inflammation by inhibiting the overactivation of CD4+, Th1, Th2, and Th17 cells and helps regulate these cells in autoimmune processes to prevent excessive immune responses [14]. Vitamin D deficiency has been associated with several autoimmune diseases, including type 1 diabetes, systemic lupus erythematosus, rheumatoid arthritis, Addison’s disease, inflammatory bowel disease, and autoimmune thyroid disease [13,14,15]. For example, a recent meta-analysis found an increased prevalence of vitamin D deficiency in individuals with HT [16], although conflicting evidence also exists [17].

HT, one of the most common organ-specific autoimmune diseases, is characterized by chronic autoimmune thyroiditis, with lymphocytic infiltration of thyroid tissue and elevated levels of anti-TPO and/or anti-TG antibodies. This leads to sustained inflammation, progressive thyroid tissue destruction, and eventual hypothyroidism [18,19,20,21]. Although the precise causes and triggers of thyroid destruction in HT remain unclear, vitamin D deficiency has been implicated in thyroid-specific inflammation and immune dysregulation due to its role in modulating immune responses [13]. Targeting modifiable risk factors, particularly vitamin D deficiency, presents a promising strategy for improving immune regulation and optimizing clinical outcomes in HT [22,23].

Vitamin D is proposed to modulate the immune response in HT through various mechanisms, including suppressing T-cell activation, regulating HLA class II expression, influencing B-cell function, and maintaining the balance between Th17 and Tregs [1]. Several studies have explored the link between vitamin D levels and antithyroid antibodies, extending the research beyond its connection to the prevalence of HT [24,25]. In a randomized controlled study by Jiang et al. [24], six months of vitamin D supplementation in HT patients reduced anti-TPO antibody levels, improved thyroid function, and slowed hypothyroidism progression. However, while some studies have identified a negative correlation between vitamin D levels and anti-TPO antibodies [18,26], others have failed to confirm this association [19,27]. Given the uncertainties in this area, our study aimed to investigate the potential associations between HT, thyroid autoantibodies, thyroid function, and vitamin D levels in a large cohort of patients.

## 2. Materials and Methods

### 2.1. Study Design

The study was conducted by retrospectively reviewing the medical records of patients aged 18 and above who were admitted to the Endocrinology and Metabolic Diseases Outpatient Clinic, Ankara Bilkent City Hospital, between February 2019 and January 2024. Participants’ age, sex, TSH, fT3, fT4, anti-TPO, anti-TG, and vitamin D levels were recorded from their most recently checked medical records.

A total of 81,180 patients were included in the study. TSH measurements were available for all participants. Among these, 15,579 had vitamin D data, 80,490 had fT4 data, 53,217 had anti-TPO data, and 54,302 had anti-TG data.

### 2.2. Laboratory Parameters

Following 8 h of fasting, fasting blood samples from routine hospital admission laboratories were analyzed. Serum TSH (mU/L), fT3 (ng/L), fT4 (ng/dL), anti-TPO (U/mL), anti-TG (IU/mL), and vitamin D (nmol/L) levels were determined by an immunassay analyzer using the chemiluminescence method with acridinium ester technology (Atellica IM 1600 Analyzer, Siemens Healthcare Diagnostics, Ankara, Türkiye).

### 2.3. Hashimoto’s Thyroiditis and Group Classification

Groups were defined as follows: HT was diagnosed based on the presence of anti-TPO and/or anti-TG antibodies. Participants with negative results for both antibodies were classified as the non-HT group. In this study, a threshold of ≥60 U/mL for anti-TPO was used to define positivity. As of 16 November 2020, the test kits utilized for the measurement of anti-TG underwent a modification. Before this date, a result of ≥60 IU/mL was deemed positive, whereas post-modification, a threshold of ≥1.3 IU/mL is now used to define a positive result.

### 2.4. Thyroid Function Status and Subgroups

The euthyroid and hypothyroid groups were defined based on TSH and fT4 levels, regardless of medication use, vitamin D supplementation, or history of surgery, as follows: individuals were classified as euthyroid if TSH was between 0.55 mU/L and 4.78 mU/L and fT4 was between 0.89 ng/dL and 1.76 ng/dL; those with TSH > 4.78 mU/L were classified as hypothyroid. Patients were further classified as euthyroid HT, euthyroid non-HT, hypothyroid HT, and hypothyroid non-HT.

### 2.5. Vitamin D Level and Classification

Patients were divided into 3 categories based on their vitamin D levels as defined by the Endocrinology Society Clinical Practice Guidelines: deficient (<50 nmol/L), insufficient (50–74.9 nmol/L), and sufficient (≥75 nmol/L) [28].

### 2.6. Statistical Analyses

Statistical analyses were performed with SPSS version 21.0 (Armonk, NY, USA: IBM Corp.). Normality of the distribution was assessed using the Kolmogorov–Smirnov test, along with histograms and Q-Q plots. All tests were two-tailed, and statistical significance was defined as *p* ≤ 0.05. Quantitative variables were reported as medians (Q1–Q3) and qualitative variables as frequencies (percentages). Categorical variables were compared between the HT and non-HT groups using the Pearson chi-square test, while numerical variables were analyzed using the Mann–Whitney U test. When more than two groups were compared, the Kruskal–Wallis test was used, followed by a step-down procedure to identify group differences. A two-step nonparametric ANCOVA method was used to compare vitamin D levels among the four groups, controlling for the effects of age and sex. Regression residuals of the ranked dependent variable were evaluated between groups using the Kruskal–Wallis test. This approach follows a similar methodology to nonparametric ANCOVA as described by Quade [29].

## 3. Results

### 3.1. Patient Demographics and Laboratory Characteristics

A total of 81,180 patients were included in the study, of which 59,001 (72.7%) were female. The median age of the patients was 51 (38–62) years. The prevalence of positive anti-TPO and anti-TG results among the participants was 31.1% and 42.2%, respectively. Additionally, 11.8% of the participants were hypothyroid, and 63.2% had vitamin D deficiency. The median value of participants’ vitamin D level was 40.93 nmol/L. Patient demographic and laboratory characteristics are summarized in Table 1.

### 3.2. Hashimoto’s Thyroiditis Status

HT status was determined based on anti-TPO and/or anti-TG results for 52,818 (65.1%) individuals. Of these, 25,018 (47.4%) did not have HT, while 27,800 (52.6%) were diagnosed with HT (Table 1).

### 3.3. Vitamin D Levels and Relationship with Hashimoto’s Thyroiditis

A statistically significant difference in vitamin D levels was observed between the HT and non-HT groups (*p* < 0.001). The median vitamin D level was 41.43 nmol/L in the HT group and 39.44 nmol/L in the non-HT group. The distribution of vitamin D status significantly differed between the HT and non-HT groups (*p* < 0.001). In the non-HT group, the proportion of individuals with vitamin D deficiency was 65.5%, compared to 62.1% in the HT group. When compared by sex, a significant difference was found in females in terms of HT and non-HT groups (*p* < 0.001). However, in males, the difference was similar (*p* = 0.215) (Table 1).

### 3.4. Thyroid Functional Status and Vitamin D Levels

The group distribution was as follows: 44.3% (n = 22,455) were euthyroid without HT, 3.1% (n = 1576) were hypothyroid without HT, 43.0% (n = 21,818) were euthyroid with HT, and 9.6% (n = 4870) were hypothyroid with HT. Among individuals with known vitamin D levels, the distribution was as follows: 40.1% (n = 4425) euthyroid without HT, 2.3% (n = 256) hypothyroid without HT, 48.8% (n = 5394) euthyroid with HT, and 8.8% (n = 971) hypothyroid with HT. Vitamin D levels differed significantly (*p* < 0.001) between four groups defined by thyroid functional status and the presence of HT. Multiple comparisons revealed the following ranking of vitamin D levels: euthyroid HT > euthyroid non-HT = hypothyroid HT = hypothyroid non-HT (Table 2).

**Figure 1:**
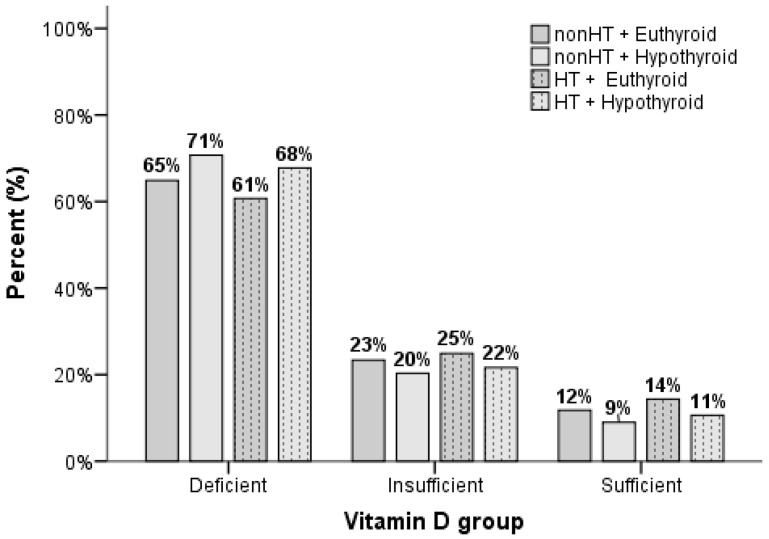
Vitamin D distribution in relation to Hashimoto’s thyroiditis and thyroid function.

The distribution of vitamin D levels in relation to HT and thyroid function is also presented in Figure 1.

### 3.5. Adjusted Analyses for Demographic Factors

After adjusting for potential confounders such as age and sex, there was a significant difference between the groups, and the ranking of vitamin D levels remained similar (*p* < 0.001, adjusted for age and sex). These results suggest that vitamin D levels are influenced by HT and thyroid function, while the effects of demographic factors persist (Table 2).

## 4. Discussion

In this study, individuals with HT had higher vitamin D levels; however, vitamin D deficiency was prevalent in both groups. Subgroup analysis revealed that the euthyroid HT group had the highest vitamin D levels, while hypothyroidism—regardless of HT status—was correlated with lower vitamin D levels. These findings indicate a potential relationship between thyroid function and vitamin D metabolism regardless of autoantibody positivity.

HT is an autoimmune disorder influenced by genetic factors and vitamin D’s role in immune function. Polymorphisms in the VDR gene may affect HT susceptibility, with some variants increasing risk while others provide protection [30,31]. Vitamin D also modulates T-cell activity, suppresses excessive immune responses, and reduces autoantibody formation, potentially preventing disease onset. Its ability to mitigate oxidative stress further complicates the relationship between vitamin D, immune function, and genetic predisposition in HT [8,18,32,33]. Kim [34] found that vitamin D insufficiency was higher in patients with autoimmune thyroid disease (AITD) compared to those without AITD (46.1% vs. 37.1%). Furthermore, patients with HT had a higher prevalence of vitamin D insufficiency compared to patients with Graves’ disease or those without AITD (*p* = 0.017). Kivity et al. [35] also found higher vitamin D deficiency in HT patients compared to those without AITD, correlating with thyroid antibodies and abnormal function tests, suggesting a role for vitamin D in AITD pathogenesis. Additionally, a meta-analysis indicated that vitamin D may reduce autoantibody titers in HT patients [36].

In line with our findings, Effraimidis et al. [37] studied women with normal TSH and thyroid antibodies in the Amsterdam AITD cohort and found no significant differences in vitamin D levels between those who developed anti-TPO positivity and those who did not over five years, suggesting no direct link between low vitamin D and early thyroid autoimmunity. Similarly, Yasmeh et al. [19] reported no association between HT and vitamin D deficiency, noting that vitamin D levels were higher in females with HT compared to those without (*p* < 0.05), while levels in males were similar.

In our study, the mean vitamin D level was lower, and the prevalence of vitamin D deficiency was higher in the non-HT group. Although there was a statistically significant difference between the groups, vitamin D deficiency was notably prevalent in both groups. It is possible that if a healthy control group without any confounding factors or comorbidities had been included, the HT group might have demonstrated a greater degree of vitamin D deficiency. However, certain underlying factors may account for this difference between the groups. One possible explanation for this finding is the possible presence of comorbid conditions in the non-HT group which are known to be associated with lower vitamin D levels, such as obesity. Another potential factor could be the higher frequency of hospital outpatient visits and subsequent vitamin D supplementation in the HT group.

Emerging evidence suggests that vitamin D may influence both immune regulation and structural maintenance of the thyroid gland. However, research exploring the link between vitamin D levels and thyroid morphology—such as gland volume, parenchymal structure, and perfusion—remains limited. Animal models suggest that the active form of vitamin D may protect thyroid tissue from structural damage and help modulate inflammatory responses [38], potentially preserving glandular integrity. Clinical evidence indicates that vitamin D deficiency in HT is associated with thyroid enlargement [25], while Grave’s disease shows an inverse correlation between vitamin D levels and thyroid volume [39]. These consistent morphological patterns across different thyroid disorders highlight the potential physiological role of vitamin D in maintaining thyroid architecture. Future studies incorporating thyroid imaging data could provide more comprehensive insights into these structural associations.

In the subgroup analysis, our study found that individuals with HT had higher vitamin D levels than those without, particularly in females, while no significant difference was observed in males. This may be due to the active immune response in HT patients, increased health consciousness leading to more vitamin D supplement use, and hormonal influences, especially estrogen. Genetic factors, dietary habits, sun exposure, and inflammation may also play roles [40]. However, although a statistical difference was identified, the vitamin D levels in both groups were found to be at deficiency levels. Consequently, a clear relationship between vitamin D deficiency and HT could not be established. Further research is essential to clarify the mechanisms and vitamin D’s potential role in thyroid autoimmunity.

In autoimmune disorders such as HT, the impaired regulatory role of vitamin D on immune responses may exacerbate autoimmune activity and lead to thyroid dysfunction. Furthermore, vitamin D deficiency is often prevalent in cases of hypothyroidism, highlighting its potential significance in thyroid pathology [41]. In this study we demonstrated that vitamin D levels were significantly lower in hypothyroid individuals. Similar to our results, in the study by Kim [34] with patients with HT and overt hypothyroidism, 25(OH)D levels were significantly lower than in euthyroid and subclinical hypothyroid patients, as well as those without AITD (80.1 ± 47.7 vs. 99.34 ± 61.2, 110.3 ± 69.9, 99.6 ± 53.7 nmol/L; *p* = 0.009). After adjusting for age, sex, BMI, and season, serum 25(OH)D levels negatively correlated with serum TSH levels (r = −0.127, *p* = 0.013). Mackawy et al. [42] demonstrated a significant association between biochemical hypothyroidism, vitamin D deficiency, and hypocalcemia, noting positive correlations between serum vitamin D, calcium levels, and thyroid hormones, alongside a negative correlation with TSH levels. These results suggest that deficiencies in serum vitamin D and calcium are linked to the severity of hypothyroidism. However, another study found no significant difference in vitamin D levels between euthyroid and hypothyroid patients with HT, with deficiency rates being similar in controls, HT, and Graves’ disease patients (25%, 23%, and 25%, respectively) [43].

If we examine the situation from a different perspective, we can speculate that hypothyroidism significantly impacts vitamin D levels, contributing to vitamin D deficiency. By slowing metabolism, hypothyroidism can hinder the synthesis, absorption, and utilization of vitamin D. Additionally, thyroid hormones play a crucial role in regulating vitamin D metabolism, thereby reinforcing the connection between thyroid function and vitamin D status. Notably, elevated vitamin D levels in HT may help sustain thyroid function.

In this study we assessed vitamin D levels at a single time point, whereas the development of autoimmunity and the onset of HT typically occur over several years. Therefore, the long-term vitamin D status of these patients remains unknown. The observation of lower vitamin D levels in the hypothyroid group compared to the euthyroid group raises the speculation that vitamin D supplementation in individuals with subclinical hypothyroidism, regardless of antibody positivity, or in euthyroid patients with positive autoantibodies might potentially reduce the rate of progression to overt hypothyroidism or delay the initiation of levothyroxine therapy. This hypothesis can be tested in further future studies.

Our study has several limitations. First, its retrospective design inherently limits the ability to establish causal relationships. Vitamin D metabolism is influenced by numerous environmental and physiological factors—including sun exposure, skin pigmentation, seasonality, sunscreen use, clothing, extracellular fluid volume, and vitamin D binding protein levels—which we were unable to fully control [44,45]. Furthermore, we did not conduct a detailed assessment of pathological or pharmacological causes of vitamin D deficiency, such as specific diseases or medication use [46]. The heterogeneity of our study population, along with unmeasured variables such as dietary habits, vitamin D supplementation, comorbidities, and smoking status, may further confound our findings [47]. Moreover, we did not evaluate metabolic characteristics such as obesity, insulin resistance, or other metabolic disorders that may influence vitamin D metabolism. In particular, the lack of data on BMI and body composition is a limitation, as obesity may reduce vitamin D bioavailability and increase its requirement [48]. We also did not assess specific nutritional deficiencies (e.g., calcium, magnesium, phosphorus), which could further impact our findings, as these nutrients are closely linked to vitamin D metabolism and function [49]. Our limited evaluation of other autoimmune conditions and thyroid dysfunctions that may influence vitamin D metabolism highlights the need for further research [50]. Although we compared patients with and without HT based on thyroid autoantibody status, the absence of a conventional control group limits the generalizability of our findings. A notable strength of our study is its large sample size.

## 5. Conclusions

In conclusion, while vitamin D deficiency was prevalent in both groups, individuals with HT showed higher vitamin D levels, whereas those with hypothyroidism exhibited lower levels. Notably, euthyroid individuals with HT recorded the highest vitamin D concentrations, suggesting that autoimmune mechanisms, alongside genetic and environmental factors, may intricately influence vitamin D metabolism. In contrast, the reduced vitamin D levels observed in the hypothyroid cohort may signify a regulatory role of thyroid hormones in this metabolic pathway. These findings underscore a compelling association between thyroid function, autoimmune processes, and vitamin D status. Nonetheless, further prospective studies are essential to elucidate a definitive cause-and-effect relationship.

## Figures and Tables

**Table 1:** The demographic and laboratory values of HT and non-HT groups.

		Total		Non-HT (n = 25,018)		HT (n = 27,800)		*p*-Value
		n	Median (Q1–Q3)	n	Median (Q1–Q3)	n	Median (Q1–Q3)	
Sex								
Male		22,179	27.3%	6859	27.4%	5307	19.1%	<0.001
Female		59,001	72.7%	18,159	72.6%	22,493	80.9%	
Age (year)		81,180	51 (38–62)	25,018	49 (36–60)	27,800	49 (38–59)	0.862
anti-TPO		53,217	-	25,018	-	27,069	-	-
Negative		36,671	68.9%	25,018	100.0%	10,523	38.9%	<0.001
Positive		16,546	31.1%	0	0.0%	16,546	61.1%	
fT3 (ng/L)		72,229	3.19 (2.89–3.50)	23,511	3.25 (2.96–3.54)	25,990	3.20 (2.91–3.50)	<0.001
fT4 (ng/dL)		80,490	1.15 (1.04–1.27)	24,923	1.15 (1.05–1.27)	27,667	1.13 (1.02–1.25)	<0.001
<0.89		4449	5.5%	1049	4.2% ^a^	1948	7.0% ^b^	<0.001
0.89–1.76		75,623	94.0%	23,764	95.3% ^a^	25,565	92.4% ^b^	
>1.76		418	0.5%	110	0.4% ^a^	154	0.6% ^a^	
anti-TG		54,302	-	25,018	-	27,451	-	-
Negative		31,374	57.8%	25,018	100.0%	4523	16.5%	<0.001
Positive		22,928	42.2%	0	0.0%	22,928	83.5%	
TSH (mU/L)		81,180	1.97 (1.28–3.13)	25,018	1.78 (1.18–2.69)	27,800	2.37 (1.47–3.90)	<0.001
0.55–4.78		72,044	88.7%	23,442	93.7%	22,930	82.5%	<0.001
>4.78		9136	11.3%	1576	6.3%	4870	17.5%	
fT4–TSH								
Euthyroid		68,475	88.2%	22,455	93.4%	21,818	81.8%	<0.001
Hypothyroid		9136	11.8%	1576	6.6%	4870	18.2%	
Vitamin D (nmol/L)		15,579	40.93 (27.46–59.65)	4816	39.44 (26.71–58.16)	6586	41.43 (27.71–60.40)	<0.001
Deficient		9843	63.2%	3153	65.5% ^a^	4087	62.1% ^b^	<0.001
Insufficient		3762	24.1%	1108	23.0% ^a^	1597	24.2% ^a^	
Sufficient		1974	12.7%	555	11.5% ^a^	902	13.7% ^b^	
Vitamin D by sex (nmol/L)	Male	4069	42.68 (30.45–59.40)	1355	42.43 (29.7–59.4)	1301	42.93 (31.2–59.65)	0.215
	Female	11,510	40.06 (26.46–59.65)	3461	38.19 (25.71–57.28)	5285	40.93 (26.71–60.9)	<0.001

n: number of patients, HT: Hashimoto’s thyroiditis, fT4: free thyroxine, ft3: free triiodothyronine, TSH: thyroid-stimulating hormone, anti-TPO: anti-thyroid peroxidase, anti-TG: anti-thyroglobulin. Data were summarized as frequencies (column percentage) for qualitative variables and medians (Quartile 1–Quartile 3) for quantitative variables. The group percentages were provided based on the known perspective variables. ^a,b^: Each subscript letter denotes a subset of group categories whose column proportions do not differ significantly from each other at the 0.05 level for more than two group comparisons. Pearson chi-square and Mann–Whitney test results were used for categorical and continuous variables, respectively.

**Table 2:** Comparison of Vitamin D levels between euthyroid non-HT, hypothyroid non-HT, euthyroid HT, and hypothyroid HT groups.

		Euthyroid Non-HT n = 4425	Hypothyroid Non-HT n = 256	Euthyroid HT n = 5394	Hypothyroid HT n = 971	*p*-Value
Vitamin D (nmol/L)		39.69 ^a^ (26.71–58.41)	36.32 ^a^ (23.77–54.35)	42.18 ^b^ (28.2–61.71)	38.19 ^a^ (25.71–55.41)	<0.001 <0.001 adjusted for age and sex *
Deficient		64.9% ^a^	70.7% ^a^	60.7% ^b^	67.8% ^a^	<0.001
Insufficient		23.4% ^a^	20.3% ^a^	24.9% ^a^	21.6% ^a^	
Sufficient		11.8% ^a^	9.0% ^a,b^	14.4% ^b^	10.6% ^a^	
Vitamin D by sex (nmol/L)	Male	42.18 (29.7–59.4)	45.68 (27.21–61.65)	42.93 (30.83–59.65)	42.18 (31.45–58.91)	0.611
	Female	38.44 ^a^ (25.96–57.78)	33.2 ^b^ (22.96–51.29)	41.93 ^c^ (27.46–62.28)	36.69 ^a,b^ (24.71–54.72)	<0.001

n: number of patients. Data were summarized as frequencies (column percentage) for qualitative variables and medians (Quartile 1–Quartile 3) for quantitative variables. The group percentages were provided based on the known perspective variables. Pearson chi-square and Kruskal–Wallis test results were used for categorical and continuous variables, respectively. Superscript letters (a, b, c) indicate group similarities based on the stepwise step-down method, based on Kruskal–Wallis test results. Each subscript letter denotes a subset of group categories whose column proportions do not differ significantly from each other at the 0.05 level, based on the chi-square test. * The analysis was conducted using a nonparametric ANCOVA, adjusted for age and sex.

## Data Availability

The data in this article will be shared at a reasonable request.

## Abbreviations

The following abbreviations are used in this manuscript:

HT	Hashimoto’s thyroiditis
TSH	Thyroid-stimulating hormone
fT4	Free thyroxine
fT3	Free triiodothyronine
25(OH)D	25-hydroxyvitamin D
1,25(OH)_2_D	1,25-dihydroxyvitamin D
VDR	Vitamin D receptor
anti-TPO	Antithyroid peroxidase
anti-TG	Antithyroglobulin
AITD	Autoimmune thyroid disease
